# Long noncoding RNA LINC01426 promotes the progression of lung adenocarcinoma via regulating miRNA-125a-5p/ casein kinase 2 alpha 1 axis

**DOI:** 10.1080/21655979.2022.2044251

**Published:** 2022-03-10

**Authors:** Xiaoling Zhu, Jianguo Zhao, Jun Xu

**Affiliations:** a Department of Oncology, Shaoxing People’s Hospital, Shaoxing, Zhejiang, China; b Department of Radiation Oncology, Shaoxing People’s Hospital, Shaoxing, Zhejiang, China

**Keywords:** Lung adenocarcinoma, LINC01426, miRNA-125a-5p, casein kinase 2 alpha 1 (CSNK2A1), malignant progression

## Abstract

Although long noncoding RNAs (lncRNAs) in lung adenocarcinoma (LUAD) have been increasingly studied, LINC01426 has not been fully investigated in LUAD. The GEPIA database revealed that LINC01426 was upregulated in LUAD tissues. In our study, we further verified the significantly high expression of LINC01426 in LUAD tissues and cell lines. We also analyzed the LINC01426 expression level and LUAD clinical features and found that high LINC01426 expression was associated with tumor diameter; tumor, node, and metastases (TNM) staging; lymph node metastasis (LNM); and overall survival (OS) rate of LUAD patients. In vitro experiments revealed that suppression of LINC01426 could repress the proliferation, migration and invasion of LUAD cells. Then, the bioinformatic analysis revealed that there were binding domains between miR-125a-5p and the 3′-UTR of LINC01426. As revealed by dual-luciferase reporter gene experiment and RNA Binding Protein Immunoprecipitation (RIP) assay, miR-125a-5p could bind to LINC01426. Additionally, the results of qRT-PCR and Pearson’s analysis respectively revealed that miR-125a-5p was slightly expressed in LUAD and its expression was negatively correlated with LINC01426. Moreover, casein kinase 2 alpha 1 (CSNK2A1) was predicted to bind to miR-125a-5p. CSNK2A1 expression was remarkably high in LUAD tissues, negatively associated with miR-125a-5p, and positively correlated with LINC01426. Subsequently, our results showed that CSNK2A1 enhanced the malignant progression of LUAD cells. Overall, our study revealed that LINC01426 might regulate the malignant phenotype of LUAD via the miR-125a-5p/CSNK2A1 axis.

## Graphical abstract

**Figure:**
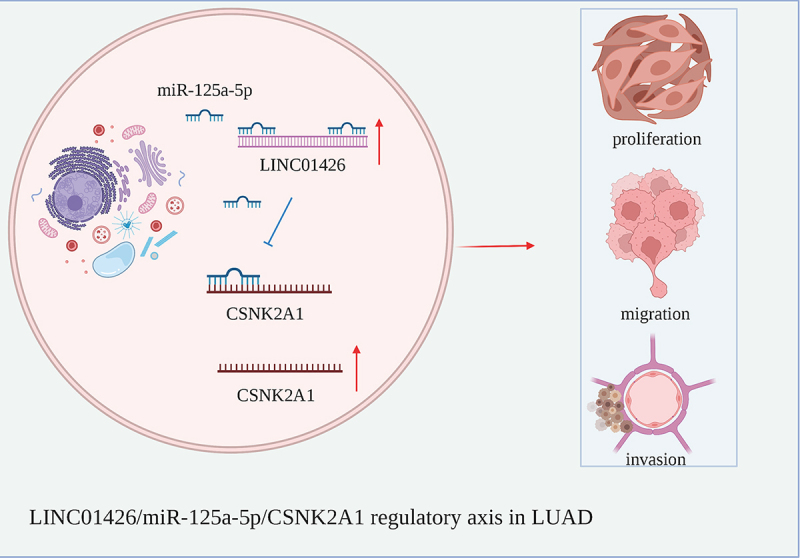

## Introduction

Lung cancer (LC) is one of the most malignant tumors with high morbidity and mortality worldwide [1]. Based on the histopathologic classification, LC can be divided into small cell LC (SCLC) and non-small cell LC (NSCLC). Lung adenocarcinoma (LUAD) is one of the primary subtypes of NSCLC, accounting for approximately 40% of all LC cases [2,3]. In recent years, with the continuous progress in medical treatment, the patients with early LUAD may typically be cured through conventional surgical procedure [4]. While those with advanced LUAD respond poorly to surgeries, with a 5-year survival rate of less than 15% [5,6]. Therefore, the potential targets and mechanism for the initiation and progression of LUAD should be explored.

Long noncoding RNAs (lncRNAs) are a class of ncRNAs with over 200 nucleotides that are incapable of encoding proteins because of the lack of an open reading frame [7]. LncRNAs are involved in the initiation and development of multiple diseases by regulating the expression of many genes. Many lncRNAs are important in various cellular processes, such as proliferation, invasion and metastasis [8]. H19 imprinted maternally expressed transcript (H19) was the first lncRNA discovered and has been shown to exert a carcinogenic or suppressive role in different tumors [9]. In recent years, the study of lncRNAs in malignant tumors has become increasingly popular. Many lncRNAs are abnormally expressed in various tumors and can act as oncogenes and tumor suppressors. For instances, small nucleolar RNA host gene 1 (SNHG1) promotes cell proliferation, migration, and invasion in cervical cancer [10]; Pvt1 oncogene (PVT1) predicts the prognosis of prostate cancer and regulates tumor growth [11]. GClnc1 can enhance the proliferation and invasion of bladder cancer by activating MYC [12]. However, the role of lncRNAs in LUAD remains to be further explored.

MicroRNAs (miRNAs) are a class of noncoding small RNA molecules of 20–24 nucleotides, regulate gene expression at the post-transcriptional level by binding to the 3′-untranslated region (3′-UTR) of target genes [13]. Previous studies have indicated that miRNAs participate in regulating multiple processes, including cell proliferation, metastasis, invasion, differentiation, cell cycle, and cell apoptosis [14]. MiR-125a-5p can exert important biological function in various malignant tumors, such as colorectal cancer [15], breast cancer [16], and gastric cancer [17]. However, the study of miR-125a-5p in LUAD only proceeds to the initial stage, warranting further studies.

LINC01426 has been shown to be involved in many tumors, such as clear cell renal cell carcinoma [18], glioblastoma [19], glioma [20] and NSCLC [21]. Besides, A study found abnormally high expression of LINC01426 in LC [22]. While the biological roles and molecular mechanisms of LINC01426 in LUAD need to be further investigated. In this study, we suggested that LINC01426 might play a vital role in the progression of LUAD. We aimed at revealing the biological functions and potential mechanism of LINC01426 regulating of LUAD progression. We first detected LINC01426 expression in the LUAD tissues and cell lines. We also identified the biological function of LINC01426 in cell proliferation, migration, and invasion of LUAD. Overall, we verified the latent mechanism by which LINC01426 might bind to miR-125a-5p and promoteCSNK2A1 expression.

## Materials and methods

### Gene expression profiling interactive analysis database

LINC01426 expression and patient prognosis were assessed using the Gene Expression Profiling Interactive Analysis database (http://GEPIA.cancer-pku.cn/index.html) [23]. This database includes 483 tumor tissue samples and 347 normal tissue samples. Using this database, correlation analyses were conducted for differentially expressed genes in tumors, including overall survival (OS) curve analysis of genes, gene co-expression analysis, correlation analysis of genes, and clinical characteristics.

### Sample collection

LUAD and normal control tissues were collected from 50 patients with LUAD. All tissues were stored at −80°C after harvesting and transported in a liquid nitrogen container for subsequent experiments. Pathological classification and tumor staging were performed according to the Union for International Cancer Control cancer staging criteria. Written informed consent was obtained from all patients before surgery. This study was approved by the ethics committee of Shaoxing People’s Hospital (Approve number: 2018–036). This study was conducted in accordance with the Declaration of Helsinki.

### Cell culture

LUAD cell lines (A549, Calu-3, H1299, and H1975) and normal human bronchial epithelial cells (HBE) employed in this study were obtained from the Shanghai Institutes of Biological Sciences, Chinese Academy of Sciences (Shanghai, China). All cells were cultured in RPMI-1640 culture medium (Thermo Fisher Scientific, Waltham, MA, USA) containing 10% fetal bovine serum (FBS; Thermo Fisher Scientific) and 1% penicillin and streptomycin. All cells were stored in an incubator containing 5% CO_2_ at 37°C.

### Cell transfection

LINC01426 specific small interfering sequence (si-LINC01426) was synthesized by GenePharma (Shanghai, China). Additionally, miR-125a-5p in vitro mimics and LINC01426 and CSNK2A1 overexpression vectors were purchased from RiboBio (Guangzhou, China). At over 60% cell adherence, cell transfection was conducted using Lipofectamine 3000 reagent (Invitrogen, Waltham, MA, USA) according to the manufacturer’s instructions [24]. All the cells were cultured in a humidified incubator with 5% CO_2_ at 37°C. The sequence of siRNAs used were: si-LINC01426 #1, sense: 5′-UUGAUAAGCUGAAAGAGACGC-3′, antisense: 5′-GUCUCUUUCAGCUUAUCAAGC-3′; si-LINC01426 #2, sense: 5′-UUUUAGUUUCUUAAUAAUGUU-3′, antisense: 5′-CAUUAUUAAGAAACUAAAAAU-3′; si-LINC01426 #3, sense: 5′-AGUUACACCGAGAUUAAACUG-3′, antisense: 5′-GUUUAAUCUCGGUGUAACUAA-3′. Transfection efficiency of more than 70% was considered as effective transfection.

### Reverse transcription quantitative real-time PCR

Total RNA was extracted from tissues or cells using TRIzol reagent (Invitrogen, Carlsbad, CA, USA). The RNA concentration was measured according to the manufacturer’s instructions. A reverse transcription kit (Takara, Shiga, Japan) was used for reverse transcription of cDNA. qRT-PCR was performed on a LightCycler®480 system (Roche) using the SYBR Green I Master reagent. GAPDH and U6 were used as internal references. The relative mRNA levels were calculated using the 2^−ΔΔCT^ method [25]. Primer sequences were as follows:

LINC01426:

F: 5′-GAACGCTTTGTCCACAGTGA-3′

R: 5′-TATCGCAGCAGTTTGTCCAG-3′

miR-125a-5p

F: 5′-GGTAAGTCACGCGGT-3′

R: 5′-CAGTGCGTCTCGTGGAGT-3′

GAPDH:

F:5′-CGGAGTCAACGGATTTGGTCGTAT-3′

R: 5′- AGCCTTCTCCATGGTGGTGAAGAC-3′

U6:

F:5′-GCTGAGGTGACGGTCTCAAA-3′

R:5′-GCCTCCCAGTTTCATGGACA-3′

### Cell proliferation assay

The cell counting kit-8 (CCK-8) (CCK-8; Dojindo, Japan) assay was performed as described previously [26]. In brief, the transfected LUAD cells were inoculated in a 96-well plate at a concentration of 4 × 10^3^ cells/well, and cultured in an incubator with 5% CO_2_ at 37°C. At 0 h, 24 h, 48 h, 72 h, and 96 h, then 10 µL CCK8 reagent was added into each well. The cells were then incubated in an incubator with 5% CO_2_ for another 2 h, followed by absorbance measurement at 450 nm.

### EdU assay

EdU assay was performed as described previously [27]. A cell-light EdU DNA kit (RiboBio, Shanghai, PR, China) was used for the EdU experiment according to the manufacturer’s instructions. A549 and H1299 cells were seeded into 96-well plates at a density of 6 × 10^3^ cells/well and processed with 50 mM EdU reagent for 2 h. Subsequently, cells were fixed in 4% paraformaldehyde and stained with DAPI. Finally, the cells were observed under an Olympus FSX100 microscope, counted, and photographed.

### Transwell assay

The transwell assay was conducted according to the manufacturer’s instructions [28]. Briefly, transfected LUAD cells were seeded into a 24-well plate containing a transwell chamber (Millipore) with an aperture of 8 nm at 4 × 10^4^ cells/well concentration. RPMI-1640 culture medium without FBS was added to the upper chamber and cells were seeded, whereas the culture medium with 10% FBS was added into the lower chamber. After 48 h of culture, the cells that did not invade the upper chamber were wiped with swabs. The cells were fixed in methanol, stained with crystal violet, imaged, counted under a microscope, and photographed. During the invasion experiment, the upper chamber was covered with substrate gel. The migration experiment was performed following the same steps, excluding the substrate gel.

### Dual luciferase reporter assay

Dual luciferase reporter assay was performed as described previously [29]. To detect the interaction of LINC01426 or CSNK2A1 with miR-125a-5p, fragments with LINC01426 or 3′-UTR wild-type binding site or mutation binding site of CSNK2A1 were inserted into the double luciferase reporter vector pmirGLO (Promega, Madison, WI, USA). Lipofectamine 3000 reagent was used to co-transfect the constructed plasmids, and miR-125a-5p mimics into LUAD cells, followed by 48 h of incubation. Luciferase activity was detected using the dual-luciferase reporter gene system (Promega) according to the manufacturer’s instructions.

### Western blotting assay

Western blotting assay was performed as described previously [30]. The transfected cells were digested and placed in a 6-well plate. After 24 h of further culture, protein expression was measured using BCA reagent. Total protein extraction and measurement were conducted using RIPA lysis buffer. In each group, 100 µg protein was subjected to electrophoresis, and a PVDF membrane was used for transfer. After 1 h of blocking at room temperature, the primary antibody was used for overnight incubation at low temperature. The next day, horseradish peroxidase-labeled secondary antibody was added, and chromogenic solution was added to develop color after 1 h of incubation, followed by photography and analysis.

### Statistical analysis

Statistical analysis were performed using GraphPad Prism 7.0. The experiments for each group were repeated three times. All data are presented as mean ± standard deviation. Student’s t-test was used for analyzing between-group differences, and Pearson’s method was used for correlation analysis. Statistical significance was set at *P* < 0.05.

## Results

This study explored the expression pattern, biological function, and latent mechanisms of LINC01426 in LUAD. We speculated that LINC01426 might play a significant role in the progression of LUAD. Our results indicate that the expression level of LINC01426 was significantly elevated in LUAD. Moreover, LINC01426 could sponge miR-125a-5p to promote CSNK2A1 expression and contribute to the malignancy of LUAD.

### LINC01426 was highly expressed in LUAD

**Figure 1.**
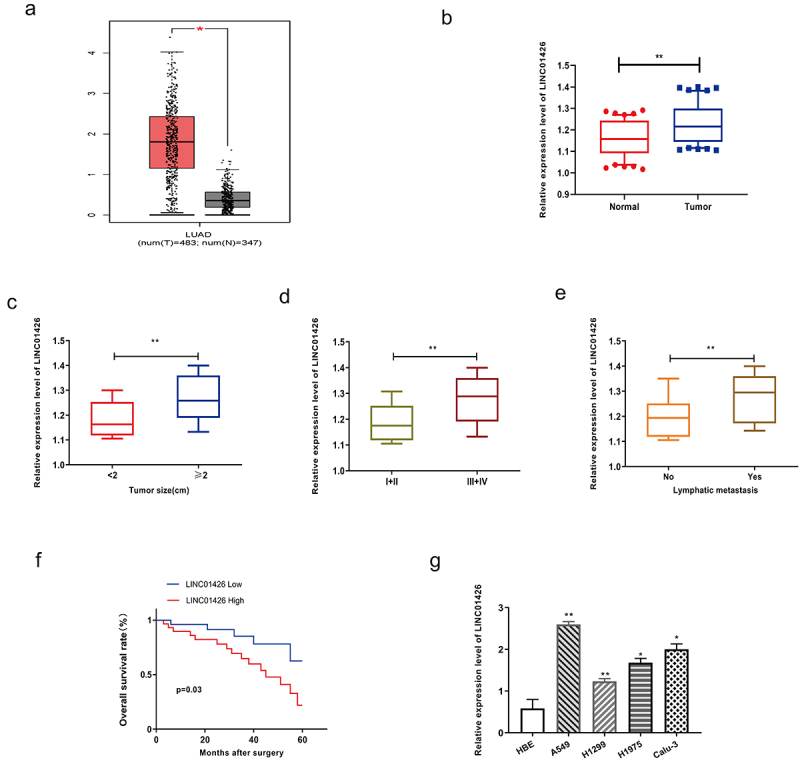
LINC01426 was highly expressed in LUAD A. The expression level of LINC01246 in LUAD tissue was analyzed by GEPIA database analysis. b. The expression level of LINC01426 in 50 pairs of LUAD tumor tissues and normal adjacent tissues was measured by qRT-PCR. c. The expression level of LINC01426 and tumor diameter were analyzed. d. The expression level of LINC01426 and tumor TNM stages were analyzed. e. The expression level of LINC01426 and lymph node metastasis were analyzed. f. The relationship between the expression level of LINC01426 and the overall survival of LUAD patients was analyzed. g. The expression level of LINC01426 in the LUAD cell line was detected by qRT-PCR. (*P < 0.05, ** P < 0.01, the expression level of LINC0142 was normalized to U6).

First, analysis of the GEPIA database revealed notably higher LINC01246 expression in LUAD tissues than in normal tissues (Figure 1(a)). Further, qRT-PCR was performed to detect LINC01426 expression levels in 50 pairs of LUAD tumor tissues and normal adjacent tissues, and found significantly higher LINC01426 expression in LUAD tissues than in normal tissues (Figure 1(b)). Subsequently, the correlation between LINC01426 expression and tumor diameter was analyzed. As shown in Figure 1(c), LUAD tissues with a tumor diameter ≥ 2 cm showed remarkably higher LINC01426 expression. Moreover, LINC01426 expression in different tumor TNM stages, LINC01426 expression in LUAD tissues in the I+ II stage was considerably lower than that in the III+IV stage (Figure 1(d)). We also observed higher LINC01426 expression in LUAD tissues with lymph node metastasis than in those without LNM (Figure 1(e)). Survival analysis indicated that LUAD patients with high LINC01426 expression had a significantly lower OS rate than those with low expression (Figure 1(f)). LINC01426 expression in LUAD cell lines was verified using qRT-PCR. As shown in Figure 1(g), LUAD cell lines (A549, Calu-3, H1299, and H1975) showed higher LINC01426 expression than the normal bronchial epithelial cells. In addition, we selected A549 and H1299 cells, which had the most significantly different expression, for subsequent experiments.

### LINC01426 knockdown inhibited the proliferation, migration, and invasion of LUAD cells

**Figure 2.**
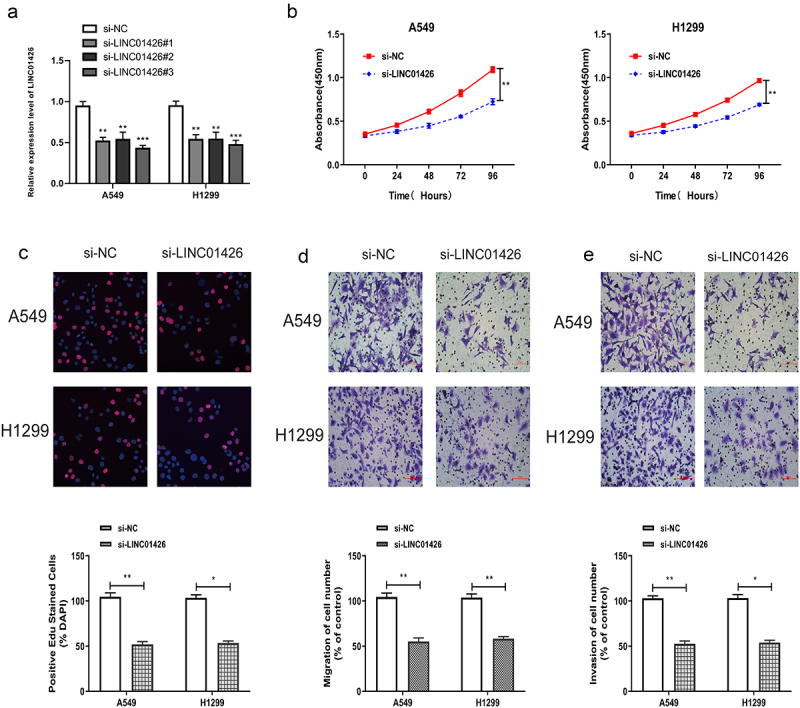
LINC01426 knockdown inhibited the proliferation, migration and invasion of LUAD cells a. The expression of LINC01426 was suppressed by small interfering RNAs (siRNAs) and the interference efficiency was confirmed by qRT-PCR. b-c. The effect of LINC01426 on cell proliferation was conducted by CCK8 and EdU experiments. d-e. The effect of LINC01426 on cell migration and invasion was conducted by transwell assay. (*P < 0.05, ** P < 0.01).

The role of LINC01426 in LUAD was verified by in vitro experiments. LINC01426 expression was suppressed by siRNA, and interference efficiency was detected by qRT-PCR. As shown in Figure 2(a), LINC01426 siRNAs could significantly suppress LINC01426 expression in LUAD cells. Among them, siRNA#3 has the best interference efficiency, so we chose it for in vitro experiments. For cell proliferation, CCK8 assay revealed that 24 h, 48 h, 72 h, and 96 h after seeding cells into 96-well plate, si-LINC01426 transfected LUAD cells had significantly lower absorbance than si-NC cells at 450 nm (Figure 2(b)). The influence of LINC01426 on the proliferation of LUAD cells was further verified by EdU assay. As shown in Figure 2(c), a significantly lower EdU-positive rate was observed for si-LINC01426 transfected LUAD cells relative to si-NC cells, suggesting that LINC01426 knockdown suppressed the proliferation of LUAD cells. We then conducted transwell migration and invasion experiments. As shown in Figure 2(d-e), remarkably less si-LINC01426 transfected LUAD cells were observed relative to si-NC, suggesting that LINC01426 knockdown suppressed the migratory and invasive abilities of LUAD cells.

### MiR-125a-5p could bind to LINC01426

**Figure 3.**
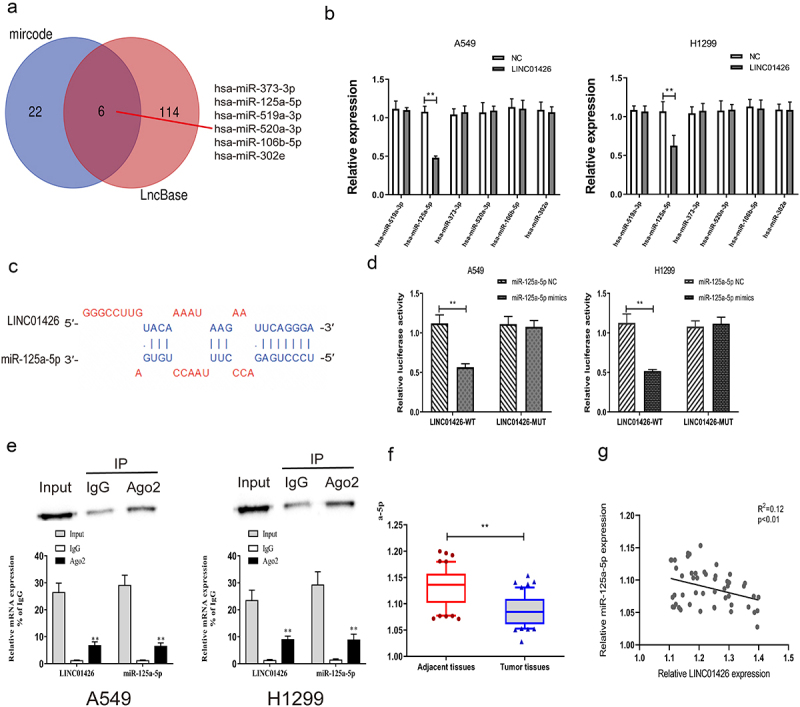
miR-125a-5p could bind to LINC01426 a. The bioinformatics websites (LncBase and mircode) were utilized to predict miRNAs that have a binding region with the 3ʹUTR of LINC01426. b. The expression levels of 6 miRNAs were analyzed in LUAD cells transfected with LINC01426 overexpression vector by qRT-PCR. c-d. The binding region of miR-125a-5p and LINC01426 was predicted through the prediction website and confirmed by dual luciferase gene report experiment. e. RIP showed the interaction between LINC01426 and miR-125a-5p in A549 and H1299 cells. f. The expression level of miR-125a-5p in LUAD tissue was measured by qRT-PCR experiment. g. The correlation between miR-125a-5p and LINC01426 expression level was analyzed by Pearson’s analysis. ** P < 0.01).

miRNAs with binding domains with the 3′-UTR of LINC01426 were predicted using bioinformatics websites (LncBase, mircode). Six miRNAs (hsa-miR-373-3p, hsa-miR-519a-3p, hsa-miR-125a-5p, hsa-miR-520a-3p, hsa-miR-106b-5p, and hsa-miR-302e) shared by these two websites were identified using a Venn diagram (Figure 3(a)). The transfection efficiency of LINC01426 overexpress plasmids (OE) was verified by qRT-PCR (Figure S1A). Subsequently, the expression of these six miRNAs in LUAD cells transfected with LINC01426 overexpression vector was detected using qRT-PCR, and miR-125a-5p with different expression levels was selected (Figure 3(b)). Several studies have identified that miR-125a-5p can act as a tumor suppressor in the initiation and development of many cancers [15,31–35]. Besides, miR-125a-5p has also been reported to repress the malignant progression of LUAD [36–38]. Hence, we selected it for further study. The binding sites of miR-125a-5p and LINC01426 were predicted using prediction websites. Then, the transfection efficiency of miR-125a-5p mimics and inhibitor was verified by qRT-PCR (Figure S1B, C). In addition, a dual-luciferase reporter gene experiment indicated that miR-125a-5p could bind to LINC01426-WT instead of LINC01426-MUT (Figure 3(c,d)). We also confirmed that LINC01426 could bind to miR-125a-5p in A549 and H1299 cells by RIP assays (Figure 3(e)). Moreover, we also constructed miR-125a-5p-WT and miR-125a-5p-MUT and transfected then with OE-LINC01426 (or OE-NC). The dual-luciferase reporter gene experiment results also identified that OE-LINC01426 could bind to miR-125a-5p -WT instead of miR-125a-5p -MUT (Figure S2). In addition, qRT-PCR experiments verified that miR-125a-5p expression in LUAD tissues was notably lower than that in normal tissues (Figure 3(f)). Pearson’s method was used to analyze the correlation between miR-125a-5p and LINC01426 expression and revealed a negative correlation for their relative expression (Figure 3(g)). Moreover, to explore the effect of miR-125a-5p on LINC01426, we transfected LUAD cells with miR-125a-5p mimics or inhibitor and detected the expression of LINC01426 via qRT-PCR. The results showed that overexpression or inhibition of miR-125a-5p could not regulate the expression of LINC01426 (Figure S3A, B).

### Overexpression of miR-125a-5p partially reversed the promotion of high LINC01426 expression on the proliferation, migration, and invasion of LUAD cells

**Figure 4.**
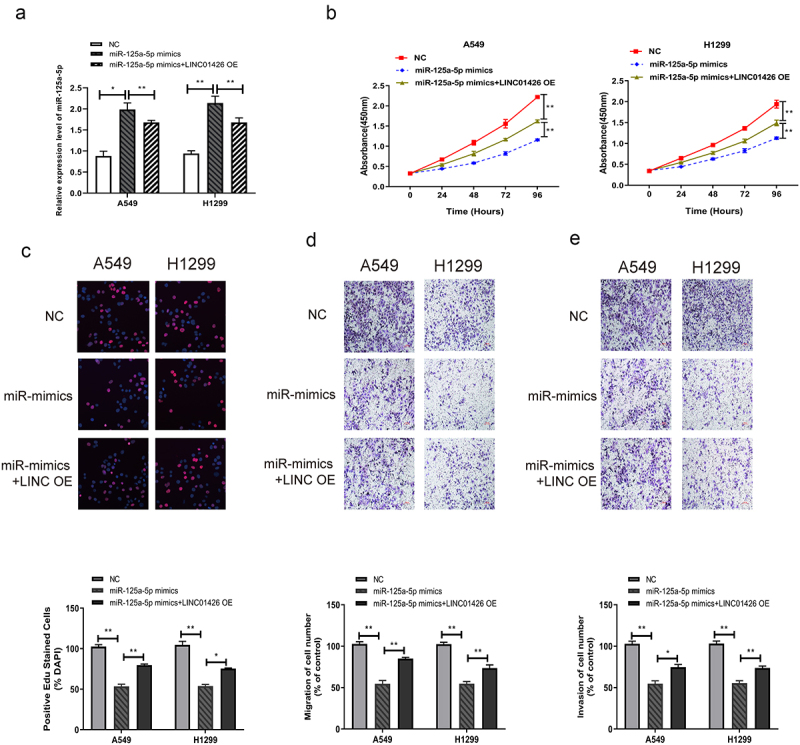
Overexpression of miR-125a-5p partially reversed the promotion of high LINC01426 expression on the proliferation, migration and invasion of LUAD cells a. The miR-125a-5p mimics and LINC01426 OE was co-transfected in LUAD cells, and the transfection efficiency was verified by qRT-PCR. b-c. The proliferation ability of miR-125a-5p and LINC01426 on cells was verified by CCK8 and EdU experiments Influence. d-e. The influence of miR-125a-5p and LINC01426 on cell migration and invasion was verified by transwell experiment. (*P < 0.05, ** P < 0.01).

To further investigate the influence of LINC01426 and miR-125a-5p on the malignant progression of LUAD, miR-125a-5p mimics and LINC01426 OE were co-transfected into LUAD cells, and transfection efficiency was confirmed by qRT-PCR. As shown in Figure 4(a), miR-125a-5p expression levels in LUAD cells were elevated after transfection with miR-125a-5p mimics, which were partially restrained by co-transfection with LINC01426 OE. The CCK8 assay revealed that transfection with miR-125a-5p mimics partially recovered the proliferation activity of LUAD cells suppressed by the transfection of LINC01426 OE (Figure 4(b)), which was further demonstrated by the EdU assay. As shown in Figure 4(c), transfection with miR-125a-5p mimics reduced the Edu-positive rate of LUAD cells by transfection with LINC01426 OE. The migratory and invasive abilities of LUAD cells were detected by transwell assay after the co-transfection of miR-125a-5p mimics and LINC01426 OE. The results indicated that the transfection of miR-125a-5p mimics partially suppressed the migratory and invasive abilities of LUAD cells transfected with LINC01426 OE (Figure 4(d,e)).

### CSNK2A1 could bind to miR-125a-5p

**Figure 5.**
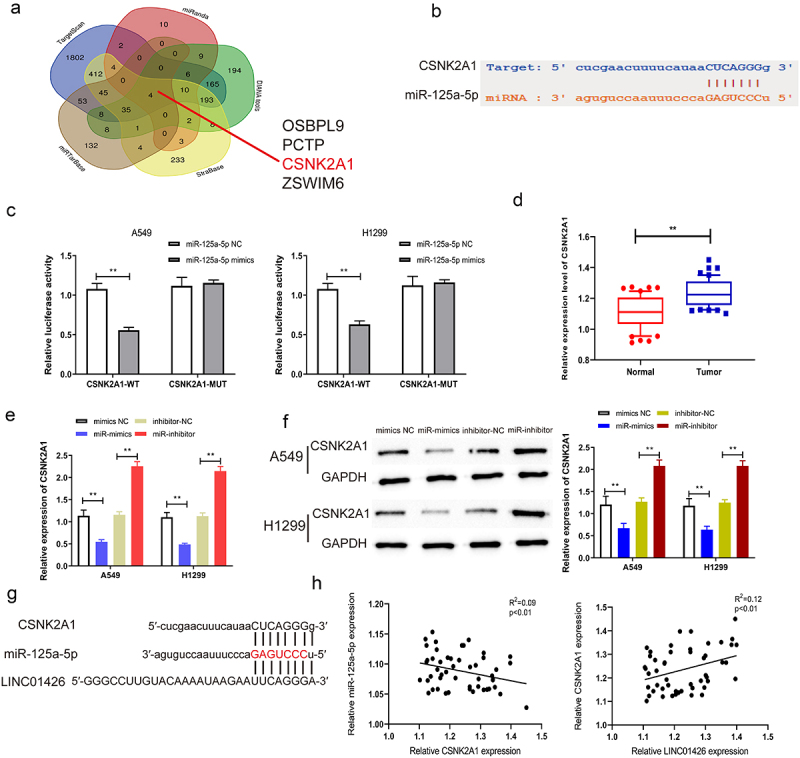
CSNK2A1 could bind to miR-125a-5p a-b. The downstream target genes of miR-125a-5p was explored through the bioinformatics website, while the CSNK2A1 exerted a higher binding score. c. The combination of miR-125a-5p and CSNK2A1 was detected through the dual luciferase gene report experiment. The binding relationship of miR-125a-5p. d. The expression level of CSNK2A1 in LUAD tissue was detected by qRT-PCR. e-f. The regulatory effect of miR-125a-5p on CSNK2A1 was detected by qRT-PCR and Western blot experiments. g-h. The correlation between CSNK2A1 and miR-125a-5p or LINC01426 expression level was analyzed by Pearson’s analysis. (** P < 0.01).

To further explore the molecular mechanisms of LINC01426 in LUAD, downstream targets of miR-125a-5p were further screened using bioinformatics; CSNK2A1 with a high binding score was screened, and their binding domains were predicted (Figure 5(a,b)). Dual-luciferase reporter gene experiments were performed to detect the binding correlation. As shown in Figure 5(c), miR-125a-5p bound to CSNK2A1-WT rather than to CSNK2A1-MUT. LUAD tissues had considerably higher CSNK2A1 expression than normal tissues based on qRT-PCR (Figure 5(d)). The association between CSNK2A1 and miR-125a-5p expression was further tested using qRT-PCR. As shown in Figure 5(e), a notable reduction in CSNK2A1 expression was observed in LUAD cells transfected with miR-125a-5p mimics, as opposed to the miR-125a-5p inhibitor. Based on the dual luciferase reporter and qRT-PCR experiments, the binding association between CSNK2A1 and miR-125a-5p was further measured by Western blotting. We identified considerably higher protein expression of CSNK2A1 in LUAD cells with the miR-125a-5p inhibitor; however, with miR-125a-5p mimics, the protein expression of CSNK2A1 was significantly suppressed (Figure 5(f)). As shown in Figure 5(g), miR-125a-5p had binding regions for both CSNK2A1 and the 3′-UTR of LINC01426. Pearson’s method was used to analyze whether CSNK2A1 expression was correlated with miR-125a-5p or LINC01426 expression. As shown in Figure 5(h), the expression level of CSNK2A1 was negatively correlated with miR-125a-5p expression, whereas a positive association was observed between miR-125a-5p and relative LINC01426 expression. These findings revealed that LINC01426 regulates CSNK2A1 expression by binding to miR-125a-5p in LUAD.

### High expression of CSNK2A1 enhanced the progression of LUAD malignant phenotype

**Figure 6.**
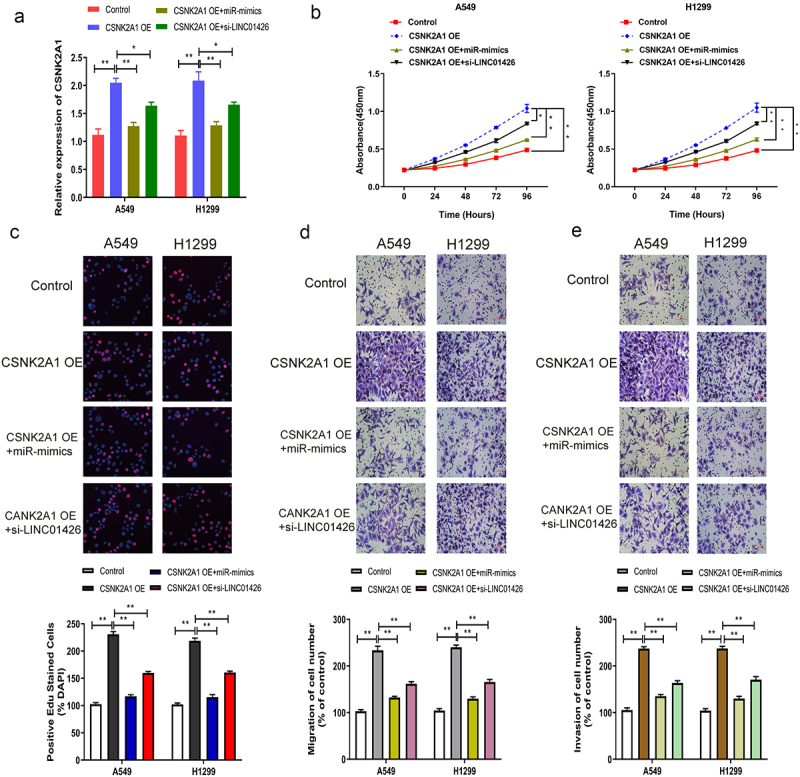
High expression of CSNK2A1 enhanced the progression of LUAD malignant phenotype a. The CSNK2A1 OE and miR-125a-5p mimics or CSNK2A1 OE and si-LINC01426 in LUAD cells were co-transfected and the transfection efficiency was verified by qRT-PCR. b-c. The effect of miR-125a-5p and CSNK2A1 on cell proliferation was detected by CCK8 and EdU experiments. d-e. The effect of miR-125a-5p and CSNK2A1 on cell migration and invasion was detected by transwell experiment. (*P < 0.05, ** P < 0.01).

The effects of CSNK2A1 on the malignant phenotype of LUAD were further investigated using several in vitro experiments. CSNK2A1 OE and miR-125a-5p mimics or CSNK2A1 OE and si-LINC01426 were co-transfected into LUAD cells, and transfection efficiency was verified by qRT-PCR. The results revealed that after miR-125a-5p mimics or si-LINC01426 were transfected into LUAD cells transfected with CSNK2A1 OE, CSNK2A1 expression was partially restrained (Figure 6(a)). Further, CCK8 and EdU assays detected LUAD cell proliferation, and the promoting effect of high CSNK2A1 expression on LUAD cell proliferation was partially suppressed (Figure 6(b,c)). Transwell, migration, and invasion experiments indicated that enhanced LUAD cell migration and invasion by high CSNK2A1 expression was partially suppressed (Figure 6(d,e)).

## Discussion

LC is the most common malignant tumor worldwide. In recent decades, with the deterioration of the global ecological environment, the incidence of LC has increased, particularly because the rising trend of LUAD is more obvious [39,40]. More than 50% of NSCLC patients progress to stage IV upon definite diagnosis, become inoperable, and suffer from poor prognosis [41]. The pathogenic factors of LC primarily include smoking, environmental factors, and genetic factors [42]. In the present study, we found that the expression of LINC01426 was abnormally high in LUAD tissues. We speculated that LINC01426 might act as an oncogene in LUAD, which was verified in a series of *in vitro* experiments.

LINC01426, also known as LincRNA-uc002yug.2, is located on chromosome 12. In a previous study, LINC01426 was shown to exert specific biological functions in glioma [20], esophageal cancer [43], and colorectal cancer [44]. A previous study reported high LINC01426 expression in LC. Corroborating the previous studies, high LINC01426 expression was observed in LUAD tissues using the GEPIA database, which was verified by qRT-PCR. Moreover, high LINC01426 expression was correlated with the clinical characteristics and prognosis of patients with LUAD. To explore the roles of LINC01426 in LUAD, a series of in vitro experiments (including CCK8, EdU, and transwell assays) revealed that high LINC01426 expression enhanced the migration, proliferation, and invasion of LUAD cells.

The seed sequence of miRNA binds to the 3′-UTR of its corresponding target gene messenger RNA (mRNA) via complementary base pairing, interferes with the normal translation process of target gene mRNA, and even degrades the target gene mRNA to activate or silence the mRNA [45]. Abnormally low miR-125a-5p expression levels were observed in LUAD tissues based on qRT-PCR, and a negative association was identified between miR-125a-5p and LINC01426 expression in correlation analysis. To further explore their combined actions on LUAD, overexpression of miR-125a-5p was found to partially rescue the promotion of high LINC01426 expression on LUAD malignant phenotype in a reversal experiment. Bioinformatics analysis revealed that CSNK2A1 is a downstream target gene of miR-125a-5p. In dual-luciferase reporter gene experiments, Western blotting, and correlation analysis, CANK2A1 was found to regulate the progression of LUAD as a target gene of miR-125a-5p. With 70 kb in length, CSNK2A1 gene consists of 13 exons, encodes 391 amino acid polypeptides, and has three conservative functional domains, including N-terminal ATP/GTP binding motif, base cluster and C-terminal activation domain [46,47]. Previous studies have revealed that CSNK2A1 is biologically important in multiple malignant tumors [^48–51^]. For instance, CSNK2A1 promotes gastric cancer invasiveness via the PI3K-Akt-mTOR signal pathway; CSNK2A1 is a mediator of MEK/ERK inhibitor resistance in KRAS (G12C) mutant LC cells [52]; CSNK2A1 is a promising new prognostic marker for renal cell carcinoma [53]. However, further research on CSNK2A1 in LUAD is required.

## Conclusions

Abnormally high LINC01426 expression was identified in LUAD, and high LINC01426 expression was related to the clinical features of patients with LUAD. LINC01426 binds to miR-125a-5p to regulate CSNK2A1, thereby regulating the proliferation, migration, and invasion of LUAD cells. This study provides new insights into the diagnosis and treatment of LUAD.

## Supplementary Material

Supplemental Material

## Data Availability

The data used to support the findings of this study are available from the corresponding author upon reasonable request.
