# Supplementary figures and images for: Long noncoding RNA LINC01426 promotes the progression of lung adenocarcinoma via regulating miRNA-125a-5p/ casein kinase 2 alpha 1 axis
Source: Bioengineered. 2022 Mar 10;13(3):7020–33. doi: 10.1080/21655979.2022.2044251 (PMC9208474; doi:10.1080/21655979.2022.2044251)

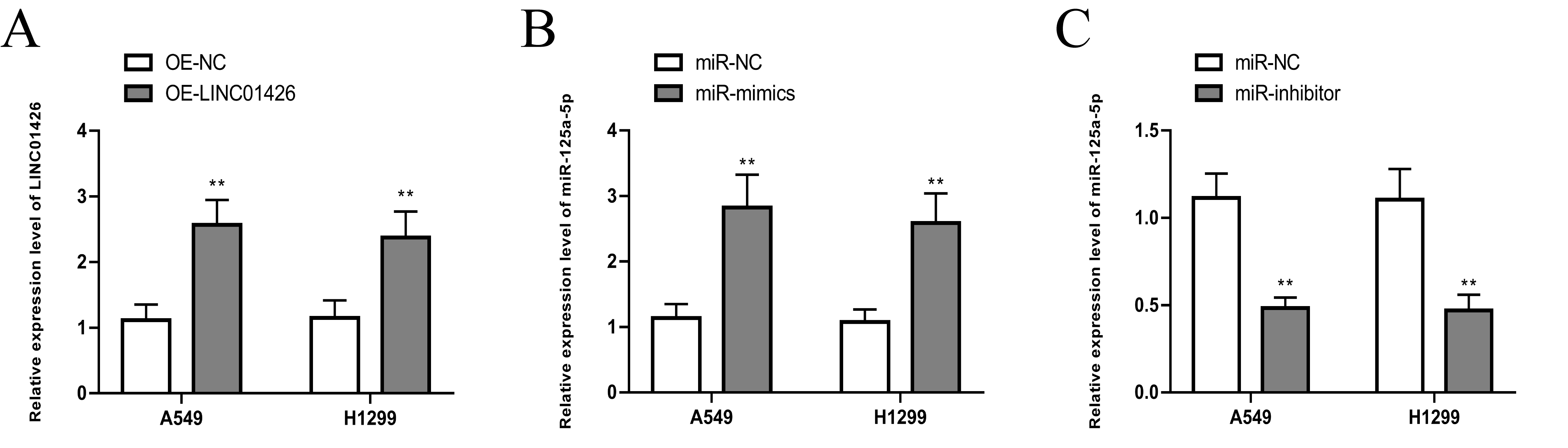

Supplement: Supplemental Material [file KBIE_A_2044251_SM9307.zip › supplementary/Figure S1.tif]

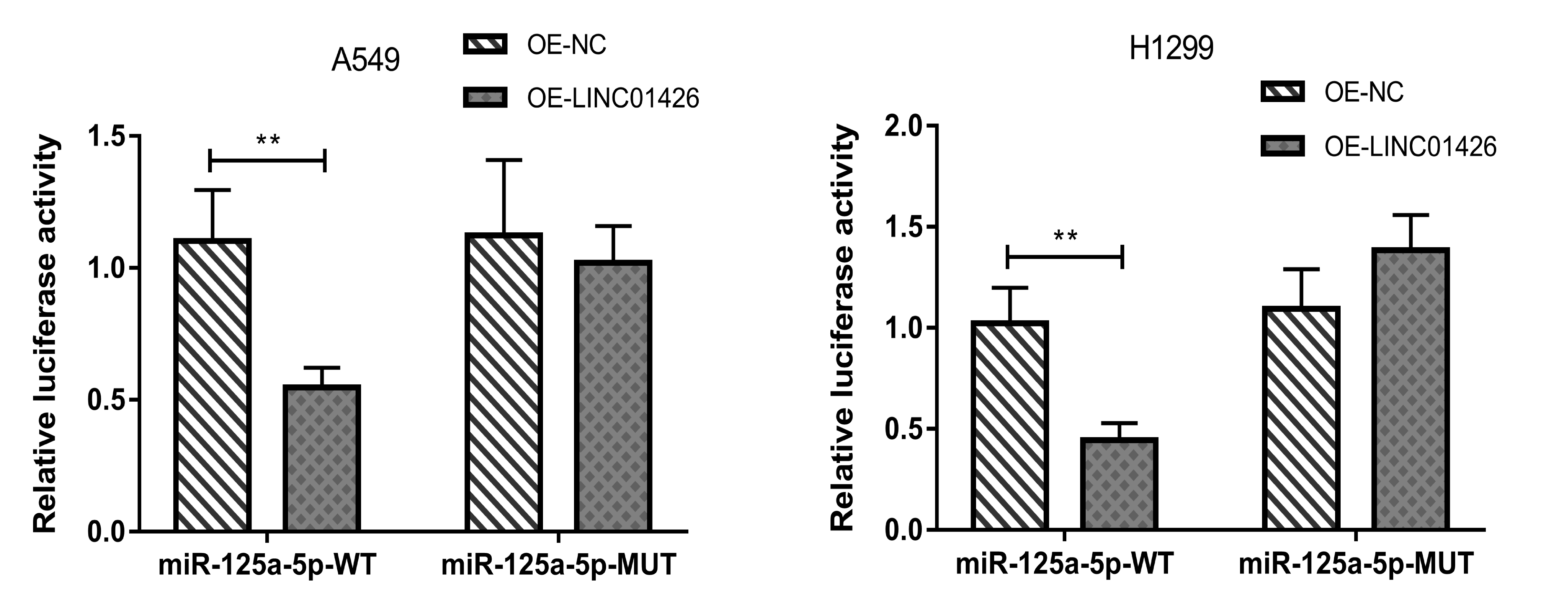

Supplement: Supplemental Material [file KBIE_A_2044251_SM9307.zip › supplementary/Figure S2.tif]

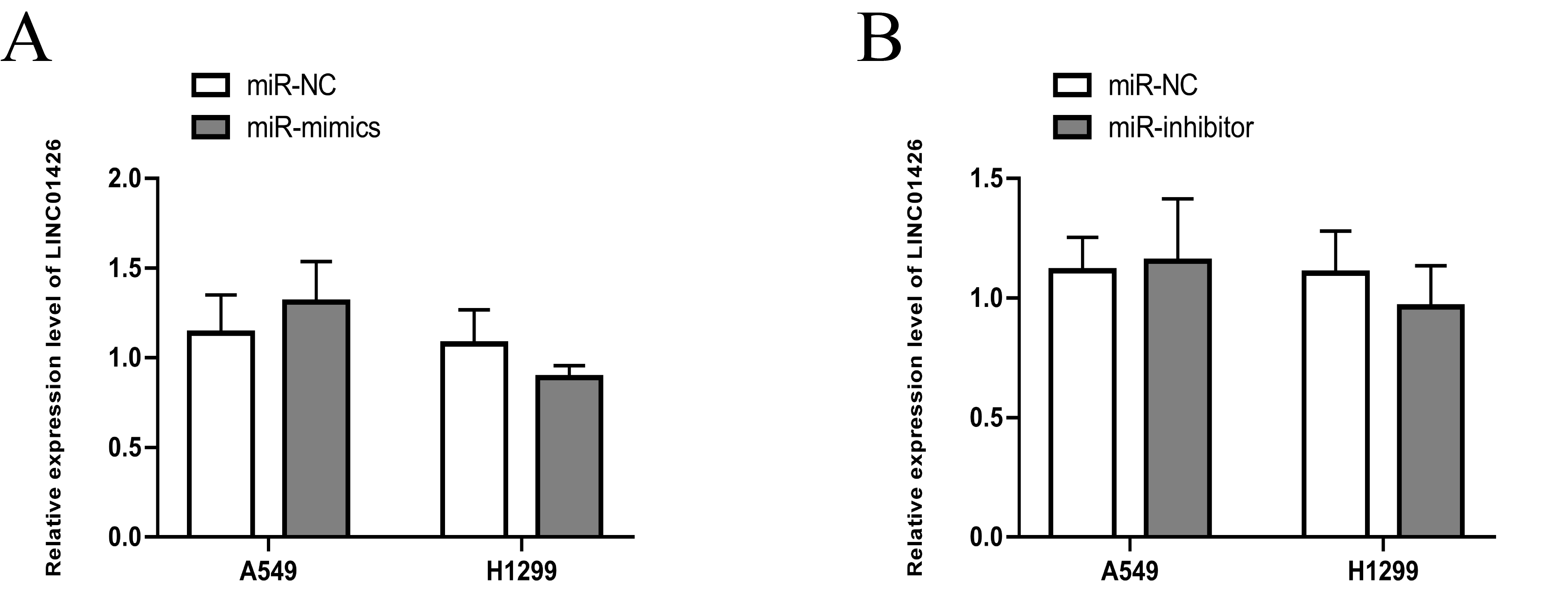

Supplement: Supplemental Material [file KBIE_A_2044251_SM9307.zip › supplementary/Figure S3.tif]
